# Acceptability and use of the electronic community health information system and its determinants among health extension workers in Ethiopia: a retrospective cross-sectional observational study

**DOI:** 10.1186/s12911-023-02385-z

**Published:** 2023-12-18

**Authors:** Tariku Nigatu Bogale, Herman Willems, Loko Abraham Bongassie, Yemariam Eyob, Chaluma Kumela Mengesha, Bantalem Yeshanew Yihun, Mesud Mohammed, Naod Wendrad, Gemechis Melkamu, Dawit Wolde Daka, Selamawit Meressa, Tadesse Alemu Bekele

**Affiliations:** 1 John Snow, Inc. (JSI), Addis Ababa, Ethiopia; 2 John Snow, Inc. (JSI), Boston, USA; 3 Emory University, Atlanta, USA; 4 Ministry of Health, Addis Ababa, Ethiopia; 5 Jimma University, Jimma, Ethiopia

**Keywords:** Acceptability, Determinant, Use, Electronic community health information system, Health extension worker

## Abstract

**Background:**

The electronic community health information system has been increasingly developed and deployed to quantify and support quality health service delivery by community health workers in Ethiopia. However, the success and failure of the electronic community health information system depend on the acceptability and use by its users. This study assessed the acceptability and use of the electronic community health information system and its determinants among health extension workers in Ethiopia.

**Methods:**

A retrospective cross-sectional observational study was conducted among 587 randomly selected health extension workers from six regions of Ethiopia. The Revised Technology Acceptance Model was used as a theoretical framework for the study. Descriptive statistics, structural equation modeling, and principal component analysis techniques were used to analyze the data. For all significance tests, multiple comparison adjustments were made using the Bonferroni Correction Method.

**Results:**

There was near universal acceptance of the electronic community health information system, ranging from 94.4 to 97.4% among health extension workers. However, actual use of the system was considerably lower, at 50%. Perceived usefulness of the electronic community health information system had a direct and positive effect on acceptability (β3 = 0.415, *p* < 0.001). Perceived ease of use had both direct and indirect positive effects on electronic community health information system acceptability (β2 = 0.340, *p* < 0.001 and β1*β3 = 0.289, *p* < 0.001, respectively), while acceptability had a direct and positive effect on the use of the electronic community health information system (β3 = 0.297, *p* < 0.001).

**Conclusions:**

Despite the very high acceptability of the electronic community health information system among health extension workers, actual use of the system is considerably lower. Hence, an integrated and coordinated approach is required to close the acceptance-use gap.

## Introduction

The World Health Organization (WHO) estimated that at least half of the world’s population cannot obtain essential health services due to a global shortage of health workers. One solution to help address this gap to train community health workers (CHWs) in low-and middle-income countries [1].

Ethiopia launched the Health Extension Program (HEP) in 2003. Health Extension Workers (HEWs) are female, trained, government-employed, and salaried frontline CHWs that implement the HEP packages. Close to 40,000 HEWs implement 18 packages of health promotion, disease prevention, and basic curative health services at the community level [2]. The HEP enabled Ethiopia to achieve significant improvements in maternal and child health, control and prevention of communicable diseases, hygiene and sanitation practices, and health care-seeking behavior [3].

A paper-based community health information system (CHIS) was designed for HEWs to capture, track, and report data about HEP services. The CHIS used by HEWs uses a family folder, a file folder that helps capture information about the household environment, socio-demographic profile, and services received in individualized cards for each family member. Using a tickler file system, a system of file arrangement enabling sequential arrangement of family folders based on appointment dates, HEWs identify defaulters and monitor individuals’ health. Health managers at different levels access the HEP data from the family folder and use it for planning, monitoring, and decision-making [4, 5].

While the CHIS has shown good report and content completeness, indicator calculation, and data display, it has problems with data accuracy and use for decision-making [6]. Generally, poor data quality is the main challenge of the health information system in Ethiopia [7, 8]. Possible explanations include the use of a paper-based system, the high cost of printing and distributing formats, the tediousness of recording and reporting formats, and user errors while recording and compiling reports [9, 10]. On average, Ethiopia spends 5,073,996.00 USD each year on reprinting health management information system formats, despite poor data quality [10]. In addition, the family folders are bulky and vulnerable to damage from rain when carried from house to house. Therefore, HEWs resort to recording in registers and transferring the data to family folders at a later time, predisposing the system to errors and poor data quality [11]. In addition, the HEP generated large amounts of data, which became manually unmanageable [4]. The family folder system also faced the challenge of redundant data elements. Time spent collecting and harmonizing redundant or non-transferable data was time that could otherwise be spent serving the community [11].

To address such challenges, Ethiopia identified information revolution (IR) as a priority agenda in its second Health Sector Transformation Plan (HSTP II) and has been implementing it since 2016. The IR, based on a strong foundation of governance, emphasizes the digitization of health information and the promotion of data use culture within the health system. In line with the IR, Ethiopia designed, developed, and implemented different digital tools, including the electronic community health information system (eCHIS) [12]. The eCHIS is a mobile-based application that works online and offline. The application is used by HEWs for household registration, service delivery, data recording, and reporting. It also facilitates referral communications between HEWs and health center staff [13]. Despite a high initial investment cost, eCHIS saves money in the long run [14], provides electronic decision support [15], facilitates faster referrals and patient tracking, minimizes duplication in recording and reporting, improves the quality of data, and enhances communication between providers, leading to improved service quality and health outcomes [16, 17]. Data accuracy, integrity, and decision-making at the community level tend to improve when using electronic CHIS (eCHIS) compared to paper-based CHIS [18]. The WHO also recommends the use of digital tools by health workers [19].

However, the benefits of digital health technology strongly depend on its acceptance and use among its end-users [20, 21]. This study assessed the acceptability and use of eCHIS among health workers in Ethiopia.

## Main body text

### Methods

#### Study design, area, and period

A retrospective cross-sectional observational study was conducted from May 15–29, 2022, in six regions of Ethiopia (Amhara, Harari, Oromia, Sidama, South Nations Nationalities and Peoples, and Southwest Ethiopia). The study was conducted among HEWs randomly selected from a representative sample of health posts in the six regions.

#### Study population and eligibility criteria

HEWs working in health posts that implemented eCHIS for at least 3 months, aged 18 years or older and who consented to participate were included in the study. HEWs who worked for less than 6 months and were not available during the data collection time were excluded from the study.

#### Sample size

The sample size was calculated using a single population proportion formula [22].

$$\mathrm n\:=\:\left[\left(\mathrm z2\ast\mathrm p\ast\mathrm q\right)+\mathrm{ME}2/\left[\mathrm{ME}2+\mathrm z2\ast\mathrm p\ast\mathrm q/\mathrm N\right]\right]/\ast\mathrm d$$

Whereas:

- n = the required sample size
- z = 1.96 at 95% confidence level
- ME = margin of error = 0.05
- p = the anticipated proportion of HEWs in the selected HPs with the attribute of interest = 50%. This assumption was made to maximize the sample size due to lack of prior studies.
- q = 1-p
- N = population size (5000)
- d = design effect =1.5

Accordingly, the calculated sample size was 537. With a 10% non-response rate, the final sample size was 591 health posts.

#### Sampling techniques and procedures

A multistage stratified random sampling technique was used to select the study subjects. First, a sampling frame of HPs that have implemented eCHIS for at least 3 months was prepared. The list was stratified by region, zone, and woreda (district). The average number of HPs that implemented eCHIS per woreda was calculated from the remaining list, which was 24. Then, the sample size was divided by this number to get the number of woredas that should be sampled to meet the sample size requirement (591/24, which equaled 25). The 25 woredas were selected from the six regions proportional to their cluster size (number of woredas per region). Once the number of woredas to be sampled from each region was determined, a table of random numbers was generated to select the 25 woredas from the six regions (Amhara 5, Harari 1, Oromia 11, Sidama 2, South Nations Nationalities and Peoples 4, and Southwest Ethiopia 2). All the health posts in the selected woredas were included in the study, and a lottery method was used to select one of the two HEWs available at the selected health posts.

#### Data collection tools

The Revised Technology Acceptance Model (TAM2) questionnaire was adapted to the local context and used in this study**.** The questionnaire has nine constructs, each of which is measured on a five-point Likert scale.

**Fig. 1:**
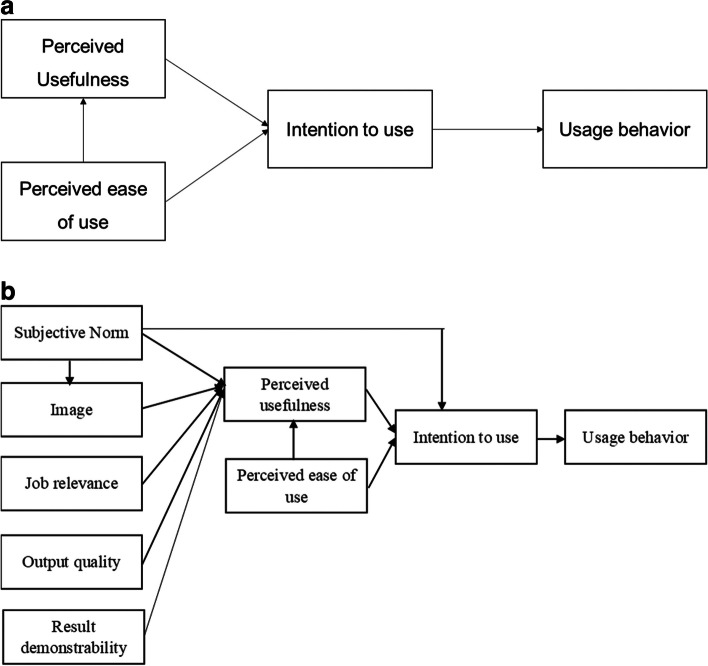
**a** Technology acceptance model (TAM) developed by Fred Davis, 1985. **b** The revised Technology Acceptance Model Two (TAM2), 2000

The TAM, based on the Theory of Reasoned Action, theorizes that an individual’s behavioral intention to use (ITU) a system (acceptability) is determined by two beliefs: perceived usefulness (PU), the extent to which a person believes that using the system will enhance his or her job performance, and perceived ease of use (PEU), the extent to which a person believes that using the system will be free of effort. According to TAM, PU is also influenced by PEU [23] (Fig. 1a).

Using TAM as the starting point, TAM2 incorporates additional theoretical constructs spanning social influence processes (subjective norm and image) and cognitive instrumental processes (job relevance, output quality, result demonstrability, and perceived ease of use) in settings where the usage of technology is mandatory. The TAM2 model has been found to be an effective model for explaining technology use intention and behavior before and during implementation [24]. Below are the definitions of the constructs used in the revised TAM2 model (Fig. 1b):

### Social influence processes

- Subjective norm (SUN): a person’s perception that most people who are important to him or her think he or she should or should not perform the behavior in question.
  a Image (Imj): is the degree to which the use of technology is perceived to enhance one’s status in one’s social system.

### Cognitive instrumental processes

- Job relevance (JR): An individual’s perception regarding the degree to which the target system is applicable to his or her job.
  a Output quality (OQ): The degree to which those tasks match end users’ job goals.
  b Result demonstrability (RD): The tangibility of the results of using the technology.

### Measurement

Items for all latent and some observed variables were measured using a five-point Likert scale: strongly disagree = 1, disagree = 2, neutral = 3, agree = 4, and strongly agree = 5. The items were asked in affirmative statements, to which respondents replied. As scoring increased from 1 to 5, it denoted a higher score in the latent and observed variables. Some items were reverse-coded to maintain this assumption.

#### Latent variables measurement

Perceived usefulness (PU) was measured using nine items. Each of the items measured different aspects of perception about eCHIS usefulness. Similarly, perceived ease of use (PEU) was measured using six items and ITU using four items. In this study, an ITU score of agree or strongly agree was used as an indicator of eCHIS acceptability.

#### Observed variable measurement

Use (usage behavior) was measured with the question: How often do you use eCHIS in your routine work? The response categories were coded as 1. Always, 2. Frequently, 3. Sometimes, 4. Occasionally, and 5. Never. In this study, use was defined as the percent of HEWs that reported used eCHIS either always or frequently in their routine work.

#### Variables of the study

The revised TAM2 model summarizes the endogenous and exogenous variables of the study. According to structural equation modeling (SEM) terminology, all boxes in the model to which an arrow is pointing are endogenous variables. All boxes with only arrows pointing away are exogenous variables.

#### Data collection procedures

The data collection tool was prepared in English and translated into Amharic and Afan Oromo languages. The translated tools were back-translated by another independent translator to check for consistency. A face-to-face interview technique was used to collect the data. A GPS-enabled Open Data Kit (ODK) was used to collect the data electronically. Experienced data collectors and supervisors were recruited and trained for 2 days. The questionnaire was checked for consistency and completeness every day during the data collection period.

#### Data processing and analysis

The data were transferred to SPSS version 25. Data cleaning was done by running frequencies and descriptive statistics. Exploratory descriptive statistics were used to understand the nature of the data. Analysis of Moment Structure (AMOS), add-in software to SPSS, was used to develop the model. A SEM was applied to test and estimate path coefficients and factor loadings. The average item score, range, and percentage of favorable item scores (4 and 5 in the Likert scale) were calculated. Increasing item scores indicate increases in the value of the underlying constructs.

Internal consistency of items was checked for the latent variables. Items with a corrected item-to-total correlation of less than 0.30 and alpha if an item deleted greater than the overall alpha were used as criteria to delete items. The dimensionality of the items was tested using principal component analysis (PCA). For all significance tests, adjustments for multiple comparisons were made using the Bonferroni Correction Method for all significant path coefficients [25].

The regression weights (β coefficients or path coefficients) and factor loadings were determined using maximum likelihood estimation (model estimation). Evaluation of model fit was made using multiple fit indices: Chi-square, Goodness of fit index (GFI), Confirmatory fit index (CFI), and values > 90 and a root mean square error of approximation (RMSEA) of value < 0.08 as cut-off points.

#### Quality assurance mechanisms

The validity of the study was ensured during data collection, entry, and analysis. Use of a standard questionnaire and electronic data collection system, translation and back translation of the data collection instruments, use of experienced data collectors, training of data collectors and supervisors, ensuring confidentiality and privacy of respondents, running descriptive statistics and frequencies, and use of advanced analysis techniques were the steps taken to ensure the quality of the study.

## Results

### Characteristics of HEWs

A total of 587 HEWs from 587 HPs in the six regions across Ethiopia were included in the study, giving a response rate of 99.3%.

The mean (+SD) age of the HEWs was 27.9 (±4.2) years. More than three-fourths (78.7%) of the HEWs were married, and the majority of them, 404 (68.8%), lived in rural areas. About two-thirds (66.6%) were trained at Level IV with a median (±IQR) year of experience of 10.0 (±9.0) years. Almost all (94.2%) HEWs received training on eCHIS. More than 70% of the HPs included in the study had two or more HEWs. Close to 80% of the HPs served 3000 or more populations in their respective catchment areas (Table 1).

**Table 1:** Socio-demographic characteristics of the HEWs, Ethiopia, June 2022

Variable	Category	Frequency	Percent
Age (in years)	20–28	367	62.5
	29–37	210	35.8
	38–46	10	1.7
Marital status	Never married	118	20.1
	Married	462	78.7
	Single (Divorced/Widowed)	7	1.2
Residence	Urban	183	31.2
	Rural	404	68.8
Education	Level I	13	2.2
	Level II	5	.9
	Level III	178	30.3
	Level IV	391	66.6
Experience (in years)	< 5	166	28.3
	5–10	137	23.3
	> 10	284	48.4
eCHIS training	Yes	553	94.2
	No	34	5.8
Number of HEWs at HPs	1	150	25.6
	2	305	52.0
	3 and above	132	22.4
Population served by HPs	< 3000	131	22.3
	3000–5000	180	30.7
	5001–7000	158	26.9
	> 7000	118	20.1

### eCHIS acceptability

The mean score of items used to measure acceptability ranged from 4.36 to 4.41 out of 5. Similarly, the acceptability of eCHIS ranged from 94.4 to 97.4%.

### eCHIS use

**Fig. 2:**
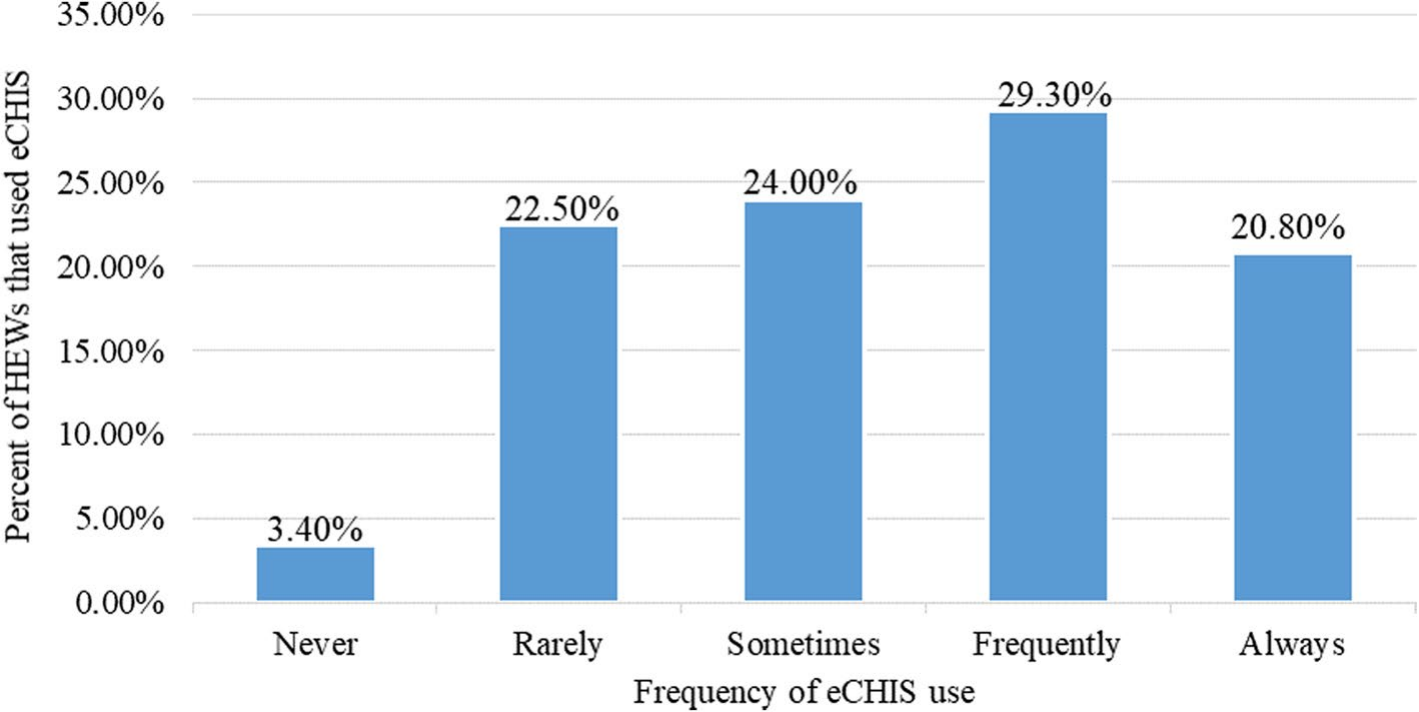
Frequency of eCHIS use among HEWs in Ethiopia, June 2022

Despite near universal acceptance of eCHIS among HEWs**,** only half, 50.1%, of the HEWs use eCHIS in their routine work (Fig. 2).

### Determinants of acceptability and eCHIS use

#### Model fitting

Initial model fitting for TAM2 did not meet cutoff points for the selected fit indices; Chi-square, GFI, CFI, and RMSEA were 4.331, 0.799, 0.799, and 0.075, respectively. Even though some improvements in the model fit indices were shown when freeing reasonable restrictions between the error terms and some latent variables, the fit indices did not meet cutoff points. Hence, the TAM2 did not replicate the sample data very well.

The TAM2 model is used in settings where the use of technology is mandatory. However, this is not the case in the current situation of eCHIS implementation, as HEWs are required to use both manual and electronic CHIS at the same time. Hence, the influence of external factors like social influence processes and cognitive instrument processes was ignored, and the TAM model was tested as a competing model. Accordingly, the TAM model demonstrated acceptable fitness for the sample data with the following values for the model fit indices: Chi-square = 3.177, GFI = 0.922, CFI = 0.935, RAMSEA = 0.06.

#### Measurement model

All except one item loaded on a single construct. Cronbach’s alpha if item deleted improved the alpha value for perceived usefulness from 0.847 to 0.879 when item nine (the advantage of eCHIS outweighs the disadvantage) was deleted from the measurement model. For the remaining constructs, there was no improvement in Cronbach’s alpha when an item was deleted. Accordingly, Cronbach’s alpha coefficients for PU, PEU, and ITU were 0.879, 0.838, and 0.863, respectively.

Confirmatory factor analysis was also done with maximum likelihood estimation to test the significance of the hypothesized measurement model. The items used to measure the constructs were all significant at a *p*-value < 0.05 (Table 2).

**Table 2:** Standardized estimates of the factor loadings of the measurement model for PU, PEU and ITU, June 2022

Code	Constructs and items	Factor loading
	**Perceived Usefulness (PU)**	
PU1	eCHIS enables me to accomplish tasks more quickly	0.642*
PU2	eCHIS has improved the quality of the service that I provide	0.673*
PU3	eCHIS has made it easier to provide health extension services	0.708*
PU4	eCHIS has improved my productivity	0.685*
PU5	I find eCHIS to be useful for my job	0.736*
PU6	Use of eCHIS increases the effectiveness of performing a task	0.731*
PU7	Using eCHIS gives me access to information I need for my work	0.686*
PU8	eCHIS provides me information for my purpose	0.694*
	**Perceived Ease of Use (PEU)**	
PEU1	My interaction with eCHIS in doing my task is clear & understandable	0.648*
PEU2	Overall, eCHIS is easy to use	0.736*
PEU3	Work with eCHIS was easy for me	0.748*
PEU4	The use of eCHIS for my daily duty does not confuse me	0.724*
PEU5	eCHIS is easy to navigate	0.702*
PEU6	Using eCHIS enables me to have more accurate information	0.517*
	**Intention to Use (ITU)**	
ITU1	I intend to continue to use eCHIS for my routine duty to perform my job	0.787*
ITU2	I intend to frequently use eCHIS for my routine duty to perform my job	0.807*
ITU3	Assuming I will have continued access to eCHIS for my routine duty, I will continue to use it	0.821*
ITU4	Given that I have access to eCHIS for my routine duty, I predict that I would adopt it	0.721*

*p < 0.001

#### Structural model

**Fig. 3:**
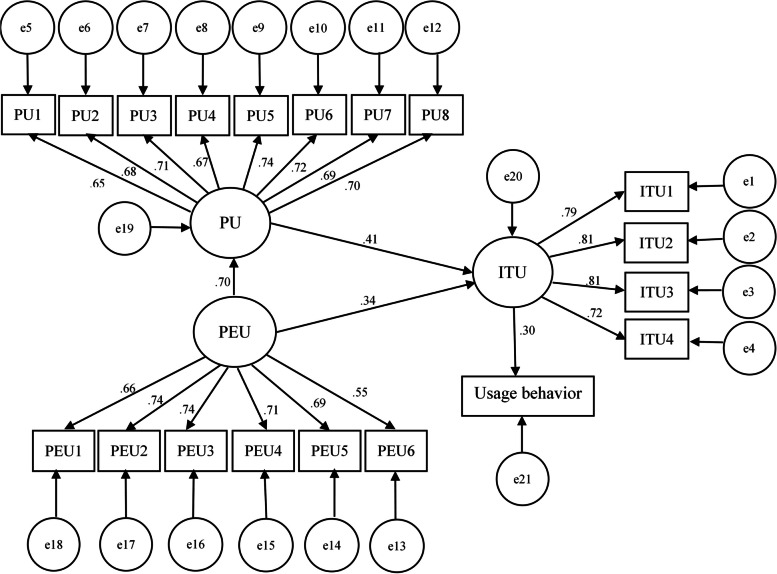
The final TAM model with standardized estimates, June 2022

The standardized path coefficients show the direction and magnitude of the association between two variables. Accordingly, ITU showed a direct and positive association with usage behavior (β4 = 0.297, *p* < 0.001). However, the strength of the association is weak, supporting the intention-use gap. Both PEU and PU showed direct and positive associations with ITU (β2 = 0.340, *p* < 0.001, β3 = 0.415, *p* < 0.001, respectively). PEU also demonstrated a significant indirect effect on ITU through its direct and positive effect on PU (β1 = 0.698, *p* < 0.001) (Table 3, Fig. 3).

**Table 3:** Standardized estimated parameters for the structural model, June 2022

Paths	Parameters	Standardized estimates
PEU→PU	β_1_	0.698*
PEU→ITU	β_2_	0.340*
PU→ITU	β_3_	0.415*
ITU→Usage behavior	β_4_	0.297*

*p < 0.001

## Discussion

This study assessed the acceptability and use of eCHIS and their determinants among community health workers in Ethiopia. This study revealed a near universal acceptability of eCHIS among HEWs. This result is in accordance with studies conducted in South Africa, Uganda, and Myanmar that reported high acceptability of digital health technologies among rural healthcare workers. Simplification of work, cost reduction, and improvement in data quality and health outcomes were reported as reasons for high acceptance by these studies [26–29].

Despite high acceptability, only half of the HEWs were using eCHIS in their routine work, demonstrating a huge acceptability-use gap. This is consistent with other studies conducted on technology acceptance among health workers in similar healthcare settings in South Africa, Uganda, Kenya, Ghana, and Brazil [26–28, 30–35]. This suggests that the high acceptability of digital technologies may not necessarily lead to high usage behavior due to barriers between acceptance and use. A study in the same setting indicated that lack of technology infrastructure, poor quality of training, lack of prior exposure to technology, workload and policy gaps hindered the use of eCHIS [36]. A well-planned and coordinated effort by the government and its partners is needed to improve use and be able to harvest the benefits from eCHIS implementation. Accordingly, careful identification and tackling of eCHIS barriers is needed, for which the MOH in Ethiopia recently developed a national eCHIS implementation strategy. The plan included major strategic shifts such as revising the eCHIS training approach, changing the eCHIS implementation design, making some policy adjustments, and strengthening the ICT infrastructure [37].

In this study, ITU (acceptability) was directly and positively, but weakly, associated with use. The weak association supports the acceptability-use gap reported in this study. A weak correlation between acceptability and use was also reported in another study that applied TAM [38]. This indicates the need to explore the barriers that hinder effective translation of use intention to actual use.

PEU showed a direct and positive effect on ITU. PEU also demonstrated a significant indirect effect on ITU through its direct and positive effect on PU. Both PEU and PU are reported to predict the acceptability of using new technology and have been used in various studies to assess technology acceptance. Similar effects were observed in various studies conducted in developing and developed countries that applied TAM to investigate technology acceptance and use [39–45]. These results may imply that, in healthcare settings, technologies that provide relevant benefits and are easy to use can be effortlessly adopted by health workers to improve their productivity. Thus, sustained effort in the development of more user-friendly eCHIS applications and hands-on training to enhance the adaptability and skills of users may lead to high acceptability and use of technology by health workers.

In this study, the concept of TAM2 was applied by considering variables that include social influence processes and cognitive instrumental processes. Nevertheless, the application of TAM2 was not replicated by the sample data. The current non-mandatory use of eCHIS by HEWs in Ethiopia may explain the lack of influence of these variables on technology acceptance and use. Hence, the effects of these variables on healthcare workers’ acceptance of new technology cannot be ruled out at this stage of eCHIS implementation. Rather, further studies should be conducted in areas where technology use is mandatory.

### Strengths and limitations

The study used a standard questionnaire to measure technology acceptability in varied contexts. In addition, data quality assurance techniques were used to generate quality data from the study. The use of advanced analysis techniques is another strength of the study. However, the study has some limitations too. This study employed a cross-sectional design. Therefore, causal links may not be established despite the use of SEM. Despite the limitations, the findings of this study advance the knowledge of eCHIS in settings where such digital technologies are used to improve the quality of service and patient outcomes.

## Conclusion

Generally, the acceptability of eCHIS is very high. However, its use for routine service delivery is considerably low. Perceived usefulness had a direct and positive effect on acceptability, while perceived ease of use had both direct and indirect positive effects on eCHIS acceptability. Acceptability had a direct and positive effect on the use of eCHIS. The acceptance-use gap may be attributed to various bottlenecks at different levels in the healthcare system. Hence an integrated and coordinated approach is required to close the acceptance-use gap by investigating and addressing use barriers.

## Data Availability

Data supporting the findings in this research article are available from the corresponding author on reasonable request.

## Abbreviations

AMOS: Analysis of Moment Structure
CFI: Confirmatory Fit Index
CHIS: Community Health Information System
CHWs: Community Health Workers
DHA: Digital Health Activity
eCHIS: Electronic Community Health Information System
GFI: Goodness Fit Index
HEP: Health Extension Program
HEWs: Health Extension Workers
ITU: Intention to Use
JSI: John Snow, Incorporated
ODK: Open Data Kit
PCA: Principal Component Analysis
PEU: Perceived Ease of Use
PU: Perceived Usefulness
RMSEA: Root Mean Square Error of Approximation
SEM: Structural Equation Modeling
TAM: Technology Acceptance Model
WHO: World Health Organization
